# Pectin lyase-modified red ginseng extract exhibits potent anti-glycation effects *in vitro* and *in vivo*

**DOI:** 10.20463/jenb.2017.0011

**Published:** 2017-06-30

**Authors:** Chan-Sik Kim, Kyuhyung Jo, Mi-Kyung Pyo, Jin Sook Kim, Junghyun Kim

**Affiliations:** 1. Korean Medicine Convergence Research Division, Korea Institute of Oriental Medicine, Daejeon Republic of Korea; 2. International Ginseng and Herb Research Institute, Geumsan Republic of Korea; 3. Department of Oral Pathology, School of Dentistry, Chonbuk National University, Jeonju Republic of Korea

**Keywords:** Advanced glycation end products (AGEs), Cross-links, GS-E3D, Red ginseng

## Abstract

**[Purpose]:**

GS-E3D is a newly developed pectin lyase-modified red ginseng extract. The purpose of this study was to evaluate the inhibitory effects of GS-E3D against advanced glycation end products.

**[Methods]:**

In this study, we evaluated the inhibitory effects of GS-E3D on the formation of advanced glycation end products (AGEs) and their cross-linking with collagen *in vitro* and in streptozotocin-induced diabetic rats.

**[Results]:**

An *in vitro* assay for the glycation of bovine serum albumin by methylglyoxal showed that GS-E3D inhibited AGE formation at an IC50 value of 19.65 ± 4.35 μg/mL. In addition, GS-E3D showed a potent inhibitory effect (IC50 = 0.42 ± 0.08 mg/mL) on the cross-linking of AGEs with collagen. However, GS-E3D showed no effect on preformed AGEs cross-linked with collagen in the breakdown assay. To determine whether GS-E3D inhibits AGE formation and their cross-linking with proteins *in vivo*, streptozotocin induced diabetic rats were treated with GS-E3D (25, 50, and 100 mg/kg/day) for 6 weeks. The administration of GS-E3D decreased serum levels of AGEs and their cross linking with proteins in diabetic rats.

**[Conclusion]:**

The inhibitory effects of this agent on advanced glycation *in vitro* and in vivo suggested that it may have a potential therapeutic role in controlling diabetes-induced AGE burden in various tissues.

## INTRODUCTION

Advanced glycation end products (AGEs) are the non-enzymati-cally modified forms of proteins or lipids generated upon exposure to sugars. AGEs form *in vivo* in hyperglycemic environments and during aging, and contribute to the pathophysiology of vascular complications in diabetes^1, 2^. 

Glycation is a spontaneous non-enzymatic reaction of free-reducing sugars with the free amino groups of proteins, DNA, and lipids, resulting in the formation of an Amadori product. The Amadori product irreversibly undergoes a variety of dehydration and rearrangement reactions, leading to the formation of AGEs. AGEs accumulate in vascular wall tissues and on plasma lipoproteins, and bind to AGE-specific receptors (RAGEs); AGEs bind to RAGE at an accelerated rate in diabetic patients, and play an important role in the development of diabetic complications^3^. Moreover, AGEs can covalently cross-link and biochemically modify protein structures, and affect the function of proteins, particularly collagen. The diabetes-induced modification of long-lived proteins, such as collagen and lens crystallin by glycation is represented an increase in the fluorescence and cross-linking of the protein^4^. The irreversible formation of AGEs and their cross-linking with proteins cause damage to the kidneys, eyes, and blood vessels^5, 6^. 

Aminoguanidine was first introduced as a AGE inhibitor^7^. As described in previous reports, aminoguanidine prevents the renal, retinal, and neural complications of diabetes through the inhibition of AGE formation^8^. However, owing to the safety concerns due to its adverse effects including pro-oxidant activities^9^ and inhibition of NO synthase^10^, aminoguanidine cannot be used clinically^11^. 

Since herbal products have generally been proven to be safer for human consumption compared with synthetic compounds, there has been an increasing interest in the use of botanical compounds as anti-AGE agents^12^. *Panax ginseng* is a widely used herbal supplement, and has been used traditionally for centuries in Asian countries to promote vitality. Red ginseng, which is the processed root of P. *ginseng* product, is manufactured through cycles of steaming and drying^13^. This manufacturing process results in the formation of additional beneficial compounds known as ginsenosides. Red ginseng has shown potent pharmacological activities against immune responses, metabolic diseases, and cancer^14-16^. Recently, some methods of transformation including enzymatic conversion^17^ and fermentation^18^ of ginsenosides from red ginseng have been used. These biotransformation processes of ginsenosides from red ginseng have increased its pharmacological potency in several animal disease models^19-22^. GS-E3D is a newly developed pectin lyase-modified red ginseng extract. This product has been shown to exhibit anti-obesity effects in a mouse model^23^, and anti-inflammatory activities in macrophage cells *in vitro*^24^. To the best of our knowledge, there have been no studies on the anti-glycation activity of GS-E3D. To address this issue, we studied the efficacy of GS-E3D as an AGE inhibitor *in vitro* and *in vivo*. In this study, the effectiveness of GS-E3D was compared with that of the well-known AGE inhibitor, aminoguanidine. 

## METHODS

### GS-E3D preparation

The material used in this experiment was a 4-yearolddried P. *ginseng* root purchased from a local market(Wooshin Industrial Co., Ltd., Geumsan, Korea), andwas deposited in the International Ginseng and HerbResearch Institute (No. GS201104). GS-E3D was preparedaccording to our previous report^23^. Briefly, redginseng extract, which was adjusted to 6° Brix wasincubated with 10% pectin lyase (EC 4.2.2.10, Novozyme, #33095, Bagsvaerd, Denmark) at 50°C for 5 daysin a shaking incubator (150 rpm). To terminate the reaction, the processed extracts were heated at 95°C for 10min, and then freeze-dried. The dried GS-E3D consistedof 120.2 mg/g crude saponin containing the followingginsenosides: 5.9 mg/g Rg1, 12.6 mg/g Re, 4.7 mg/gRf, 30.2 mg/g Rb1, 14.0 mg/g Rc, 17.6 mg/g Rb2, 2.5mg/g Rb3, 27.7 mg/g Rd, 1.3 mg/g 20(S)-Rg3, 1.4 mg/g20(R)-Rg3, 0.8 mg/g Rk1, and 1.5 mg/g Rg5. 

### Inhibitory effect on AGE formation *in vitro*

AGEs were produced in the *in vitro* system by a mo-dified method that has been previously described^25^. Bovine serum albumin (10 mg/mL, Sigma Chemicals, MO, USA) was incubated at 4°C for 7 days with methylglyoxal (5 mM) in sodium phosphate buffer (0.1 M, pH 7.4). All of the reagents and samples were sterilized by filtration through 0.2 mm membrane filters. This reaction mixture was then mixed with GS-E3D. Aminoguanidine (Sigma Chemicals, MO, USA) was used as a positive inhibitor. The fluorescence intensity of fluorescent AGE formation was measured using a spectrofluorometric detector (BIO-TEK, Synergy HT, Ex: 350 nm, Em: 450 nm). The concentration of each test sample resulting in 50% inhibition of the activities (IC_50_) was estimated from the least squares regression line of the logarithmic plot of concentration against the remaining activity. 

### Inhibitory effect on AGE cross-linking

The ability of compounds to inhibit AGE cross-linking was measured by a previously reported method^26^. Briefly, the mixture of 1 μg AGE modified bovine serum albumin (BSA) (Cosmo Bio, Tokyo, Japan) with either test concentrations of GS-E3D or aminoguanidine was added to each well of collagen-coated microtiter plates (Sigma, MO, USA). AGE-BSA was allowed to react with collagen for 4 h at 37°C. The formation of the collagen-AGE-BSA complex was detected using an anti-AGE monoclonal antibody (6D12, Cosmo Bio, Tokyo, Japan), a horseradish peroxidase-conjugated goat anti-mouse IgG antibody, and a H_2_O_2_ substrate containing ABTS chromogen. The optical density (OD) at 410 nm was measured on an ELISA reader (BIO-TEK, synergy HT). The inhibition of cross-linking was expressed as the percentage decrease in OD when AGEBSA was incubated with collagen in the presence of the compounds. 

### Cross-link breaking effect on preformed AGE cross-links

The ability of GS-E3D to break preformed AGEs was measured by a previously reported method with minor modifications^27^. Briefly, 1 μg of glycated bovine serum albumin (AGE-BSA, MBL International, MA, USA) was preincubated in collagen-coated 96-well plates (Nunc, Roskilde, Denmark) for 24 h, and the collagen-AGE-BSA complexes were incubated with or without GS-E3D (Sigma, MO, USA) or alagebrium (Suchem Pharma Co., Wenzhou, China). The collagen-AGE-BSA cross-linking was detected using an mouse anti-AGE antibody (6D12, Wako, Osaka, Japan), a horseradish peroxidase-linked anti-mouse IgG antibody, and a substrate containing 3,3′,5,5′-tetramethylbenzidine chromogen. The levels of cross-link breakage were calculated as the percentage decrease in optical density at 410 nm. We calculated the inhibitory concentration 50% (IC_50_, μg/mL) as 50% inhibition of the collagen-AGE-BSA cross-linking. 

### Animals

Seven-week-old male Sprague-Dawley rats were purchased from Orient Bio (Seongnam, Korea), and acclimated for 1 week prior to the study. Diabetes was induced by a single injection of streptozotocin (STZ, 60 mg/kg, i.p.). The age-matched control rats received an injection of an equal volume of vehicle (0.01 M citrate buffer, pH 4.5). One week after the STZ injection, a blood sample was obtained from the tail vein. Rats with a blood glucose level over 300 mg/dL were defined as diabetic rats. The rats were randomly divided into 5 groups of 10 each as follows: (1) normal control rats (NOR), (2) STZ-induced diabetic rats (DM), and (3, 4, and 5) STZ-induced diabetic rats treated with GS-E3D (25, 50, and 100 mg/kg body weight, respectively). GS-E3D was dissolved in the vehicle (distilled water). GS-E3D was orally administered to the rats for 6 weeks. All experimental procedures were performed under the supervision of our Institutional Animal Care and Use Committee (IACUC approval No. 15-100). 

### Quantification of AGE formation in vivo. 

To determine AGE formation, serum samples were analyzed by a competitive enzyme-linked immu-nosorbent assay (ELISA). The assay was performed using a monoclonal AGE antibody (6D12, Cosmo Bio, Tokyo, Japan) according to established protocols^28^. 

### RBC-IgG assay

Immunoglobulin G (IgG) is observed to be cross-linked to the membrane proteins of red blood cells (RBCs). RBC-IgG complexes are formed before other AGE cross-links *in vivo*. The amount of RBC-IgG can be used to estimate the levels of protein cross-linking^27^. To test the inhibitory effect of GS-E3D on AGE cross-linking, RBCs from heparinized whole blood were collected, and RBC-IgG levels were determined using anti-IgG ELISA. 

### Statistical analysis

The results were evaluated statistically using oneway analysis of variance, followed by the Tukey’s multiple comparison test using GraphPad Prism 4.0 (Graph-Pad Software, San Diego, CA, USA). 

## RESULTS

### Inhibitory effect of GS-E3D on AGE formation in vitro

GS-E3D was analyzed by *in vitro* bioassays to eva-luate AGE-BSA formation. The inhibitory effect of GS-E3D on AGE-BSA formation is summarized in Table 1. GS-E3D inhibited the formation of AGE-BSA (IC_50_ = 19.65 ± 4.35 μg/mL). The inhibitory activity of GS-E3D was stronger than that of aminoguanidine (IC_50_ = 80.28 ± 3.39 μg/mL) and the unmodified red ginseng extract (IC_50_ = 139.46 ± 68.18 μg/mL). 

**Table 1.** Inhibitory effects of GS-E3D on AGE formation.

Agent	Half-maximal Inhibitory Concentration (IC_50_)
GS-E3D	19.65 ± 4.35 μg/mL
Red ginseng extract	139.46 ± 68.18 μg/mL
Aminoguanidine	80.28 ± 3.39 μg/mL

The inhibitory effect is expressed as the mean ± S.D. of triplicate experiments. The IC_50_ values were calculated from the dose-inhibition curve.

### Inhibitory effect of GS-E3D on the cross-linking of AGEs with collagen in vitro

**Figure 1.**
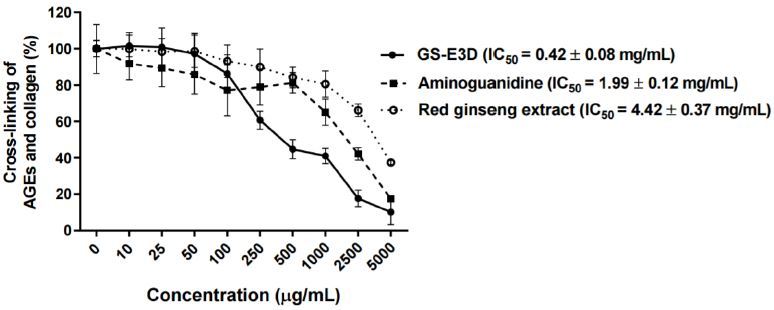
Inhibitory effect of GS-E3D on the cross-linking of AGEs with collagen *in vitro*. The cross-linking of AGE-BSA with collagen was detected by ELISA. Data are presented as means ± SE (n = 4). The IC_50_ value was calculated from the dose-inhibition curve. Aminoguanidine was used as the positive control.

The inhibition of the cross-linking of AGE-BSA withcollagen at various concentrations of GS-E3D was tested(Figure 1). GS-E3D decreased the cross-linking of AGE-BSA with collagen in a dose-dependent manner; the IC_50_value of GS-E3D was 0.42 ± 0.08 mg/mL, and its inhibitoryactivity was stronger than that of aminoguanidine (IC_50_ valueof 1.99 ± 0.12 mg/mL) and the unmodified red ginsengextract (IC_50_ = 4.42 ± 0.37 μg/mL). 

### Effect of cross-link-breaking of GS-E3D on preformed AGE cross-links with collagen in vitro

**Figure 2.**
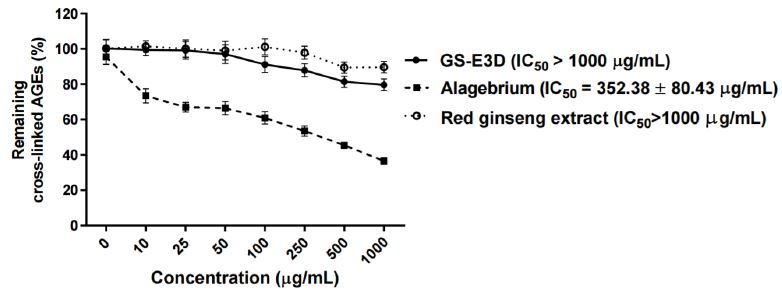
Effect of cross-link-breaking of GS-E3D on the preformed cross-links of AGEs with collagen *in vitro*. The cross-linking of AGE-BSA with collagen was detected by ELISA. Data are presented as means ± SE (n = 4). The IC_50_ value was calculated from the dose-inhibition curve. Alagebrium was used as the positive control.

We tested whether GS-E3D could also interact with preformed AGEs *in vitro*. As shown in Figure 2, incubation with GS-E3D, the unmodified red ginseng extract or alagebrium over a range of concentrations destroyed the preformed AGE-BSA-collagen cross-links. Alagebrium, which is a well-known AGE-breaker, dose-dependently destroyed the cross-links in the preformed AGE-BSA complexes with rat-tail tendon collagen (IC_50_ = 352.38 ± 80.43 μg/mL). However, GS-E3D and the unmodified red ginseng extract did not break the cross-links of AGE with collagen. 

### Blood glucose

Blood glucose levels are summarized in Table 2. Blood glucose levels had significantly increased in the diabetic rats (p < 0.05). No differences in blood glucose levels were observed between the GS-E3D-treated and vehicle-treated diabetic rats. 

**Table 2.** Blood glucose levels

		NOR	DM	GS-E3D (mg/kg)		
				25	50	100
Blood Glucose (mmol/L)	Initial	3.52 ± 0.34	17.07 ± 1.82*	17.07 ± 2.40	17.08 ± 2.54	16.74 ± 2.23
	Final	3.79 ± 0.82	18.61 ± 5.16*	18.19 ± 8.11	18.36 ± 4.21	19.41 ± 2.41

NOR, normal rat; DM, STZ-induced diabetic rat; GS-E3D, DM treated with GS-E3D (25, 50, or 100 mg/kg). All data are expressed as means ± standard deviation (n = 10); *p < 0.05 vs. NOR group.

### GS-E3D inhibits AGE formation in vivo and AGE cross-linking

**Figure 3.**
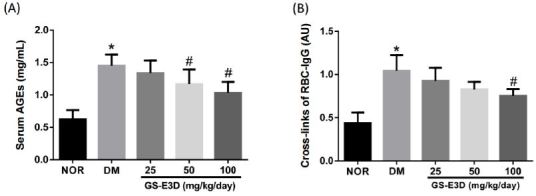
Effects of the *in vivo *treatment with GS-E3D on AGE formation (A) and IgG cross-linking with the RBC surface (B) in the blood of the streptozotocin-induced diabetic rats. The values in the graph represent means ± SE (n = 10). *p < 0.05 vs. normal control rats, #p < 0.05 vs. vehicle-treated.

The ability of GS-E3D to inhibit AGE formation *in vivo* was tested. At the end of the study, the AGE levels in serum were remarkably elevated in the vehicle-treated diabetic rats compared to the normal control rats. However, these levels in the GS-E3D-treated diabetic rats had considerably decreased compared to the vehicle-treated diabetic rats (Figure 3A). Next, we carried out an RBC-IgG assay to evaluate AGE cross-linking. As shown in Figure 3B, the RBC-IgG level in the vehicle-treated diabetic rats had substantially increased compared to that in the normal control rats. However, treatment with GS-E3D considerably reduced the level of RBC-IgG compared to the vehicle-treated diabetic rats. 

## DISCUSSION

Many dietary supplements are being sold with advertisements of their numerous beneficial effects. GS-E3D is a commercial pectin lyase-modified red ginseng extract containing a high level of the ginsenoside Rd. In the present study, we demonstrated that the newly developed pectin lyase-modified red ginseng extract, GS-E3D had an inhibitory effect on AGE formation and the cross-linking of AGEs with collagen *in vitro* and *in vivo*. 

It is well established that AGE formation plays a crucial role in the development of diabetic complications^29^. The AGE cross-links are permanent, and irreversible complexes are formed when glucose binds to the target protein, such as collagen. The cytotoxic roles of AGEs in diabetes have been shown in a number of studies^30^. AGEs can accumulate in many tissues of patients with diabetes. Since the body does not contain any enzyme capable of structurally degrading the AGEs, they accumulate during the biological life of the proteins^31^. 

There is considerable interest in agents that inhibit the formation of AGEs and their cross-links or those that can break the AGE cross-links due to their therapeutic potential^6, 32^. Several synthetic and natural agents have been proposed as AGE inhibitors. Reactive carbonyl species are potent precursors in the formation of AGEs and cross-linking of proteins^33-36^. AGE inhibitors, including aminoguanidine and pyridoxamine prevent AGE accumulation by interacting with the reactive carbonyl species and acting as carbonyl traps^37^, ^38^. However, owing to safety concerns, aminoguanidine is not currently used^11^. Recently, several researchers have suggested that a novel agent can destroy preformed AGE-derived protein cross-links. The first AGE breaker to be identified, PTB was introduced in 1996. Since PTB is unstable *in vitro*, it was not clinically successful. Another compound, alagebrium^39^ was developed as an AGE breaker. Alagebrium could reverse AGE accumulation *in vivo*^40^. Since the clinical studies on these compounds were terminated, none of the known AGE breakers is in clinical use. 

Our previous studies showed that some natural herbal products have potent anti-AGE activities^41, 42^. Quan et al. reported that Korean red ginseng reduced the formation and secretion of AGEs in the kidneys of diabetic rats^43^. The ginsenoside Rd is one of the bioactive compounds present in red ginseng, and it ameliorated the damage to astrocytes induced by methylglyoxal, which is a precursor of AGEs^44^. Since GS-E3D has an high level of Rd compared with an unmodified red ginseng extract^45^, GS-E3D may exert a more potent inhibitory effect than the unmodified red ginseng extract on the formation of AGEs and their cross-linking with proteins. Although GS-E3D has potent inhibitory effects on AGE formation *in vitro* and *in vivo*, the mechanism of its action is still not clear. Based on our findings, the inhibition of the formation of AGEs and their cross-links with proteins by GS-E3D might ameliorate the AGE burden in the diabetic rats. Furthermore, these data support the premise that GS-E3D is effective for the treatment of AGE-related diabetic complications due to the inhibition of AGE formation in various tissues and in the serum. 

In conclusion, our study showed that GS-E3D is a potent inhibitor of the formation of AGEs and their cross-linking with proteins. Although we did not compare the effects of GS-E3D with those of an unmodified red ginseng extract in the animal model, GS-E3D has more potent anti-AGE activity than the unmodified red ginseng extract *in vitro*. Therefore, GS-E3D could be a promising drug candidate for the treatment of AGE-related diseases by reducing AGE burden. 
